# A Novel Method for Measuring the Pupil Diameter and Pupillary Light Reflex of Healthy Volunteers and Patients With Intracranial Lesions Using a Newly Developed Pupilometer

**DOI:** 10.3389/fmed.2021.598791

**Published:** 2021-09-07

**Authors:** Joji Kotani, Hiroyuki Nakao, Isamu Yamada, Atsushi Miyawaki, Naomi Mambo, Yuko Ono

**Affiliations:** ^1^Department of Emergency, Disaster and Critical Care Medicine, Hyogo College of Medicine, Nishinomiya, Japan; ^2^Division of Disaster and Emergency Medicine, Department of Surgery Related, Kobe University Graduate School of Medicine, Kobe, Japan

**Keywords:** mobile type pupilometer, digital record, direct and indirect irradiation, pupil diameter, pupil light reflexes, miosis, mydriasis, intracranial lesions

## Abstract

**Background:** Physicians currently measure the pupil diameter and the pupillary light reflex with visual observations using a ruler and a traditional penlight, leading to possibly inaccurate and subjective assessments. Although a mobile pupillometer has been developed and is available in clinical settings, this device can only assess one pupil at a time. Hence, an indirect pupillary light reflex, including those under irradiation to the opposite side of pupil, cannot be evaluated. Consequently, we have developed a new automatic mobile pupilometer, the Hitomiru^®^, with Hitomiru Co., Ltd. (Tokyo, Japan). This device is a two-glass type pupilometer with a video recording system. The pupil diameter and light reflex of both pupils can be measured simultaneously; therefore, both indirect and direct light reflexes can be assessed.

**Purpose:** To evaluate the clinical ability of the Hitomiru^®^ pupilometer to assess the pupil diameter and the pupillary light reflex of healthy volunteers and patients with intracranial lesions in an intensive care unit (ICU).

**Methods:** Twenty-five healthy volunteers and five ICU patients with intracranial lesions on only the left side were assessed using the Hitomiru^®^ pupilometer. The protocol was as follows: infrared light was applied to both pupils, followed by visible light to the right pupil, infrared light to both pupils, visible light to the left pupil, and then infrared light to both pupils. All the intervals were 2 s, and the dynamics of pupil diameters on both sides were continuously recorded.

**Results:** The healthy adults had approximately 0.5 mm anisocoria, miosis was harder, and mydriasis was less with increased age. There were several differences in miosis rates, miosis times, and mydriasis rates between the healthy adults and the patients with intracranial lesions with both direct irradiation and indirect irradiation.

**Conclusions:** The initial trial estimated and digitally recorded direct and indirect light reflexes, including rapidity of miosis after direct and indirect lights on, and mydriasis after direct and indirect lights off. The Hitomiru^®^ pupilometer was a useful device to digitally record and investigate the relationship between pupil reflexes and intracranial diseases.

## Background

Prompt assessment of the pupil function in the clinical settings, such as during pre-hospital emergency care, in the emergency room, intensive care unit (ICU), or operation room, is critical to the evaluation of neurological function of critically ill patients who may have intracranial lesions. However, physicians currently measure the pupil diameter and the pupillary light reflex with visual observation using a ruler and a traditional penlight, leading to inaccurate and subjective assessment values [(1), Wilson (2), p. 897], and resulting in no digital records. This situation is in contrast to other vital sign recordings, such as information regarding respiratory and circulatory conditions that are evaluated and recorded digitally.

Although a mobile pupilometer has been developed and is available in the clinical setting (3–9), this device can only assess one pupil at a time. Hence, an indirect pupillary light reflex cannot be evaluated. Abnormal pupillary light reactivity such as time extension of light reflex is seen in the patients with intracranial disease such as increased intracranial pressure (2). In addition, evaluation of direct and indirect pupillary light reflex is necessary for differential diagnosis of optic nerve injury, oculomotor nerve damage, brain stem lesions, such as tumors, and medications (10) although the presence or absence of the difference between direct and indirect light reflex and the meaning of such difference in the patients with intracranial lesions have not been elucidated yet. Consequently, we have developed a new automatic mobile pupilometer, the Hitomiru^®^ pupilometer, which is a two-glass type pupilometer incorporated with a video recording system, in cooperation with the University of Tokyo, Hyogo College of Medicine and Hitomiru Co., Ltd.

The Hitomiru^®^ pupilometer has two characteristics: (1) the pupil diameter and light reflex of both pupils can be measured simultaneously; therefore, both indirect and direct light reflexes can be assessed, (2) the time of start of both miosis after direct and indirect lighting on, and mydriasis after direct and indirect lighting off, can be measured, (3) all the data are recorded digitally, leading to accurate and objective assessments, and (4) the instrument is a mobile type pupilometer, which can be easily transported to an ICU, emergency room, and pre-hospital care field.

The purpose of this study was to assess the pupil diameter and the direct and indirect pupillary light reflex in healthy volunteers using Hitomiru^®^ pupilometer and investigate the relationship between these measurements and age because aging may influence neuro-activity involved in pupillary light reflex, and to investigate the differences in these parameters measured by Hitomiru^®^ pupilometer between volunteers and patients with intracranial lesions in an ICU. This is the first trial to estimate and digitally record direct and indirect light reflexes, including the rapidity of miosis after direct and indirect lighting on, and mydriasis after direct and indirect lighting off, simultaneously.

## Materials and Methods

The study was approved by the Institutional Review Board of Hyogo College of Medicine. It was conducted from January 2015 to October 2015 using healthy volunteers, and patients in the emergency ICU of Hyogo College of Medicine Hospital, which is a 20-bed maximum care unit.

### Study Subjects

The healthy volunteers were included in the study after informed consent was received from them. They were 20–91 years of age (47.3 ± 18.7 years). The number of individuals (25 total) included five in each age group (in their teens, twenties, thirties, forties, fifties, and over 60 years of age), and consisted of 15 males and 10 females. The healthy volunteer group had neither intracranial lesions nor eye lesions, except for eyesight drops although they did not check precise ophthalmologic examinations. All of volunteers did not have past history, comorbidity, and medication that affect neurological response including pupil light reflex.

The patients in our ICU were included in the study after informed consent was received from the patients or their legal guardians. Inclusion criteria included the existence of intracranial lesions located on only the left side, to avoid confusion in the analysis of the results. The exclusion criteria included the existence of eye lesions such as cataract except for eyesight drop, past history like as diabetes mellitus that influenced the speed of pupil light reflex, preexistent pupillary abnormalities, active malignant disease, a do-not resuscitate decision, and/or refusal of study inclusion by the patient or the guardian or consent given too late for study inclusion. There were five patients, consisting of three males and two females. The ages ranged from 48 to 89 years (74.0 ± 14.5 years). All the patients except one were not given medicine that affects pupil light reflex including propofol, midazolam, and fentanyl. The patient characteristics, which included age, sex, disease type, treatment regimen, Glasgow Coma Scale, Factors that affect neurological response including past history, comorbidity, and medication, sedative and analgesics, midline shift on head CT, and time to measure from onset of the disease, are shown in Table 1.

**Table 1:** Patients' characteristics.

**No**	**Age**	**Sex**	**Disease**	**Lesion side**	**Treatment regimen**	**Glasgow coma scale**		**Factors that affect neurological response**			**Sedative and analgesics**	**Midline shift on head CT**	**Time to measurement from onset**
						**Initial**	**Worst**	**Past history**	**Comorbidity**	**Medication**			
1	75	F	Middle cerebral artery occlusion	Left	Thrombectomy	4 5 6	3 4 6	None	None	None	None	None	Day 2
2	86	M	Lobar hemorrhage	Left	Endoscopic drainage	4 5 6	4 5 6	None	DM	None	None	None	Day 2
3	72	M	Middle cerebral artery occlusion	Left	Thrombectomy	1 1 1	1 T 1	None	DM	None	Midazolam (10 mg /h)	None	Day 2
4	47	F	Middle cerebral artery occlusion	Left	Conservative treatment	1 2 4	1 T 4	None	None	None	Fentanyl citrate (0.037 μg/kg/min)	None	Day 2
5	89	M	Acute subdural hematoma	Left	Conservative treatment	3 5 5	3 5 5	None	None	None	None	None	Day 3

### Measurement Mechanism of the Hitomiru^®^ Pupilometer

**Figure 1:**
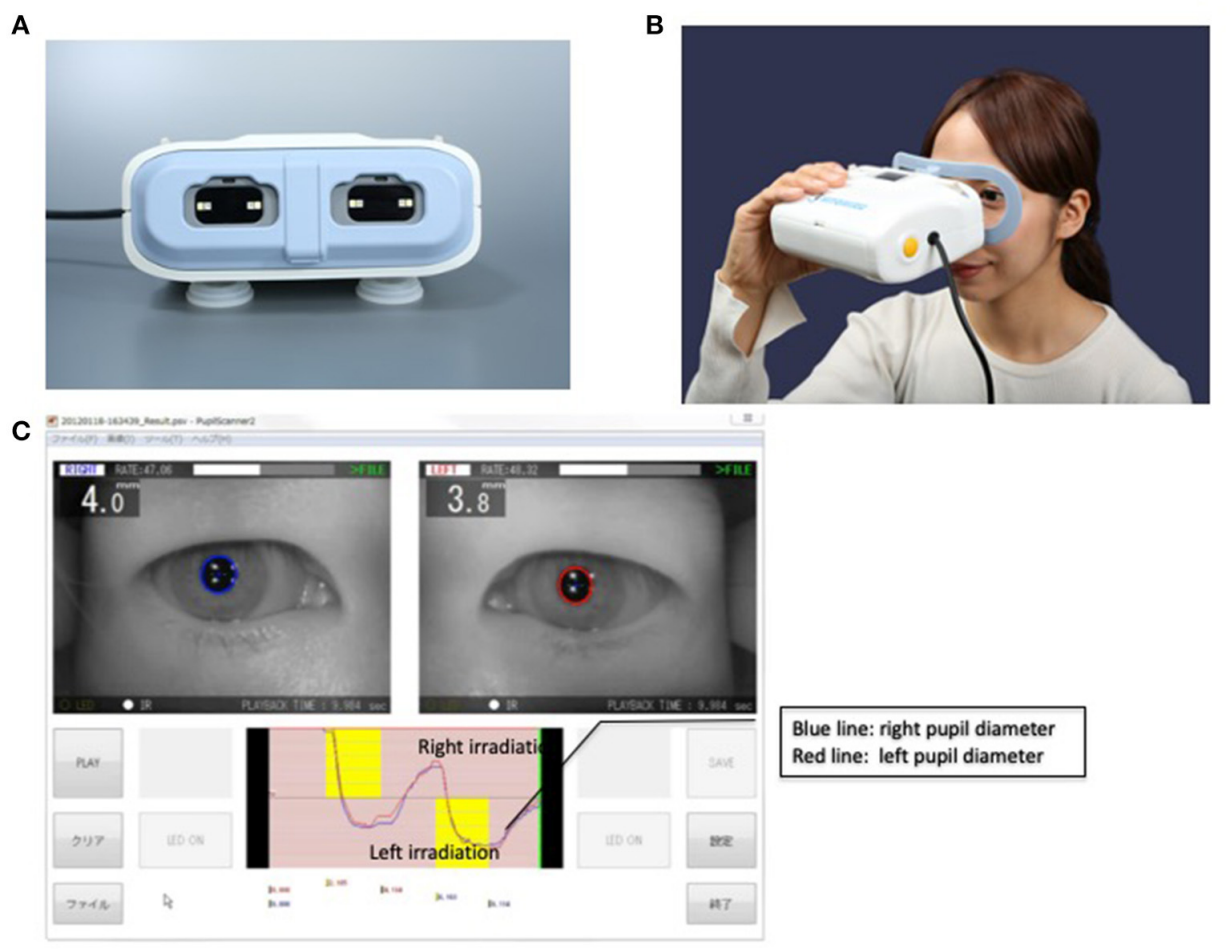
The Hitomiru^®^ pupillometer. **(A)** The shape of the Hitomiru^®^ device is like a glass with light sources and a video camera which allows automatically captures the image of pupils. The captured data is sent to the computer, which is connected to the pupillometer, then the diameters are recorded and analyzed with included proprietary software. **(B)** The Hitomiru^®^ pupillometer used on a volunteer. **(C)** Measurement screen of the device. In the upper image, the right and left pupil is captured with a blue and red circle, respectively. In the lower image, the blue and red line shows a right and left pupil diameter, respectively. The yellow zone in the upper and lower section of the graph shows the right and left eye irradiation, respectively. When the irradiation induces miosis, the lines go downward. Conversely, when the cessation of irradiation induces a mydriasis, the lines go upward.

The Hitomiru^®^ pupilometer evaluated the pupils with video recordings, regardless of the state of consciousness. When the patients could not open their eyes with obeying to the order because of depressed level of consciousness, we opened their eyelids by ourselves. Visible light was utilized to estimate the reactivity to light and infrared wavelength, which did not stimulate pupil reactivity, was often utilized to measure and monitor the size or diameter of a pupil. The Hitomiru^®^ pupilometer shined visible light onto one side of the pupil to induce miosis, and then used infrared light to measure and monitor the size or diameter of both pupils by using infrared short-range sensors. Infrared short-range sensors were also used to gauge the distance between the camera and pupil to compensate for the measured diameter of each pupil. The boundary of each pupil was detected automatically. The area of each pupil was estimated and expressed as the number of pixels. The diameter of each pupil was regarded as a straight line that passed through the center of the pupil. The diameter of the pupil was calculated using the numerical value of the diameter and the coefficient of the distance between the camera and pupil (Figure 1). One session consisted of one 10-s interval. The detailed protocol was as follows: (1) infrared light exposure to both pupils for 2 s, (2) visible light exposure to the right pupil for 2 s, (3) infrared light exposure to both pupils for 2 s, (4) visible light exposure to the left pupil for 2 s, and (5) infrared light exposure to both pupils for 2 s.

### Study Protocols

We evaluated three parameters of the direct and indirect pupillary light reflex; the miosis rate (the minimum pupil diameter during light irradiation/the maximum pupil diameter before light irradiation), the miosis time (the time until minimum pupil diameter during light irradiation), and a mydriasis rate (the minimum pupil diameter during light irradiation/the maximum pupil diameter until 2 s after discontinuation of light irradiation, which was usually a pupil diameter at 2 s after discontinuation of light irradiation). The measurements were performed with the Hitomiru^®^ pupilometer under room light using a fluorescent lamp with a mean illumination of 1,049–1,133 lux during the daytime.

#### Study 1

We measured the pupil diameter and the direct and indirect pupillary light reflex of the right and left pupils of 25 healthy adult volunteers. The right pupil diameter and direct and indirect pupillary light reflexes of the left pupils were determined.

#### Study 2

The same measurement procedures were performed on 25 healthy adult volunteers (the same volunteers of Study 1). The data were analyzed for correlations between the measurement data (pupil diameter, and direct and indirect pupillary light reflex) and age.

#### Study 3

The same measurement procedures were used for the patients with intracranial lesions. The patients' pupil reflexes were measured when we obtained informed consent from the patients' family and these were Day 2 or Day 3.

The data were compared with that of the volunteer group (measurement of the pupil diameter and pupillary light reflex). For the purpose of integrating the average age of the volunteer group and the patient group, we choose five subjects who were over 60 years of age among the 25 volunteers. All data compared the five volunteers with five patients.

## Statistical Analyses

**Figure 2:**
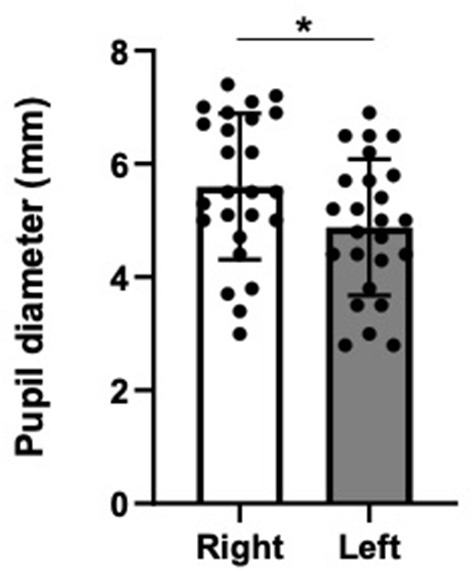
Spontaneous pupil diameters without irradiation in healthy volunteers. Data are presented as the mean ± SD. Closed circles show data distribution. ^*^P < 0.05. *N* = 25/group.

**Figure 3:**
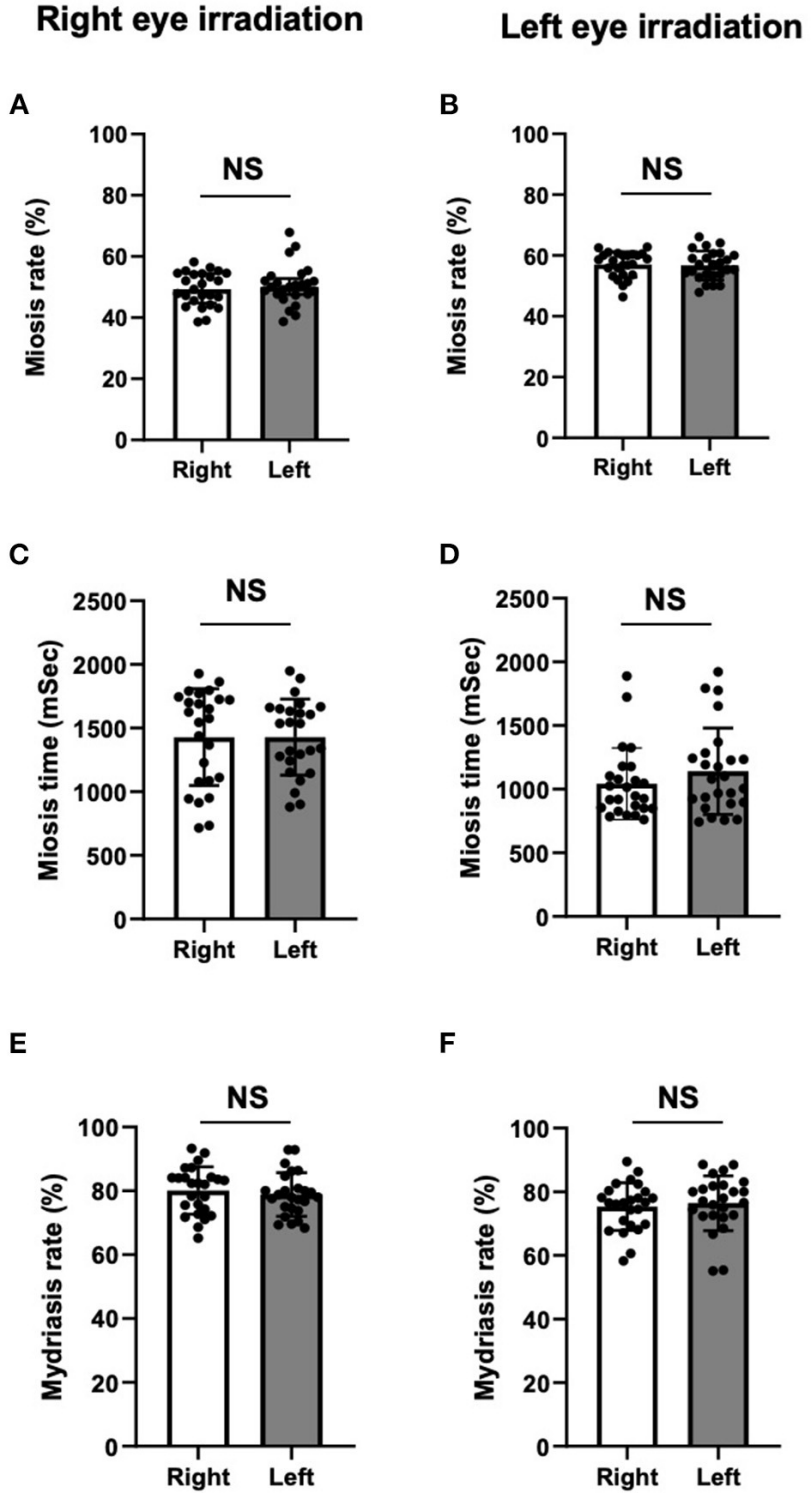
Miosis rates **(A,B)**, miosis times **(C,D)**, and mydriasis rates **(E,F)** in healthy volunteers. There were no significant differences in miosis rate **(A,B)**, miosis time **(C,D)**, and mydriasis rate **(E,F)** in both right and left pupils between direct and indirect irradiation. For all panels, Data are presented as the mean ± SD. Closed circles show data distribution. NS, not significant. *N* = 25/group.

**Figure 4:**
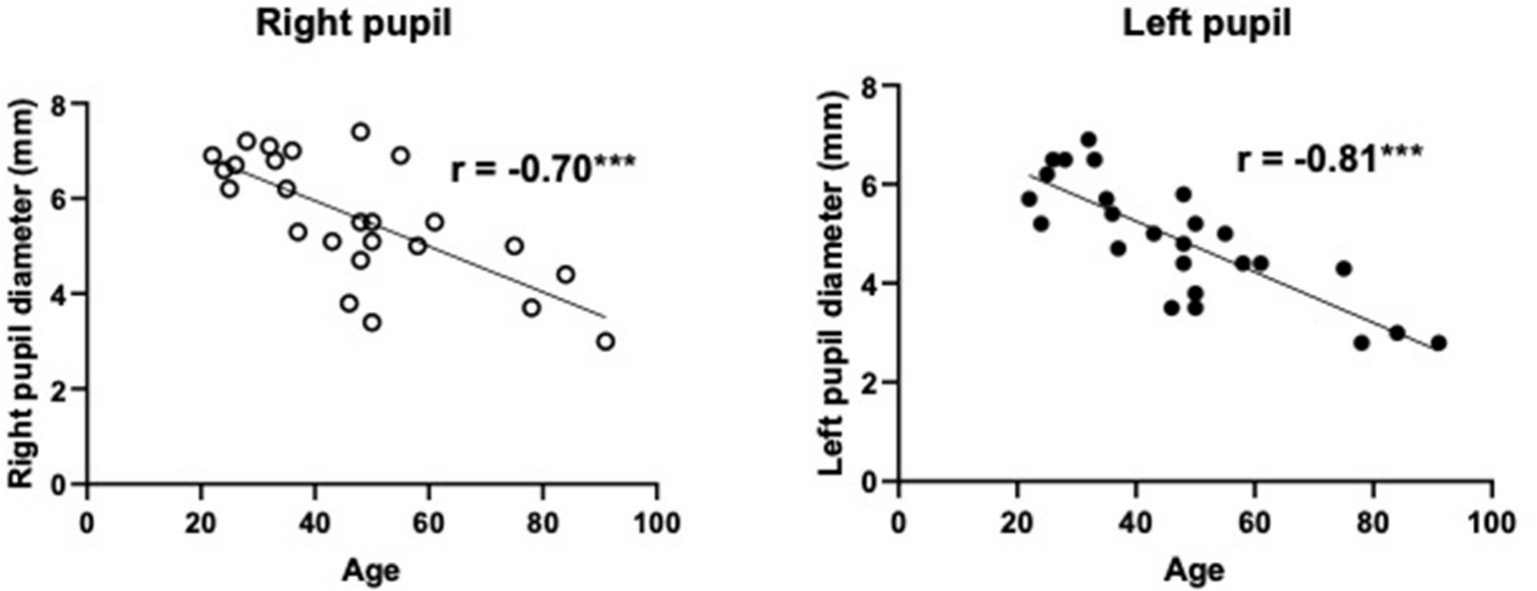
The relationship between spontaneous pupil diameters and age in healthy volunteers. The pupil diameters were significantly smaller with increased age in both the right and left eyes. *r*, correlation coefficients. ^***^P < 0.001. *N* = 25/group.

**Figure 5:**
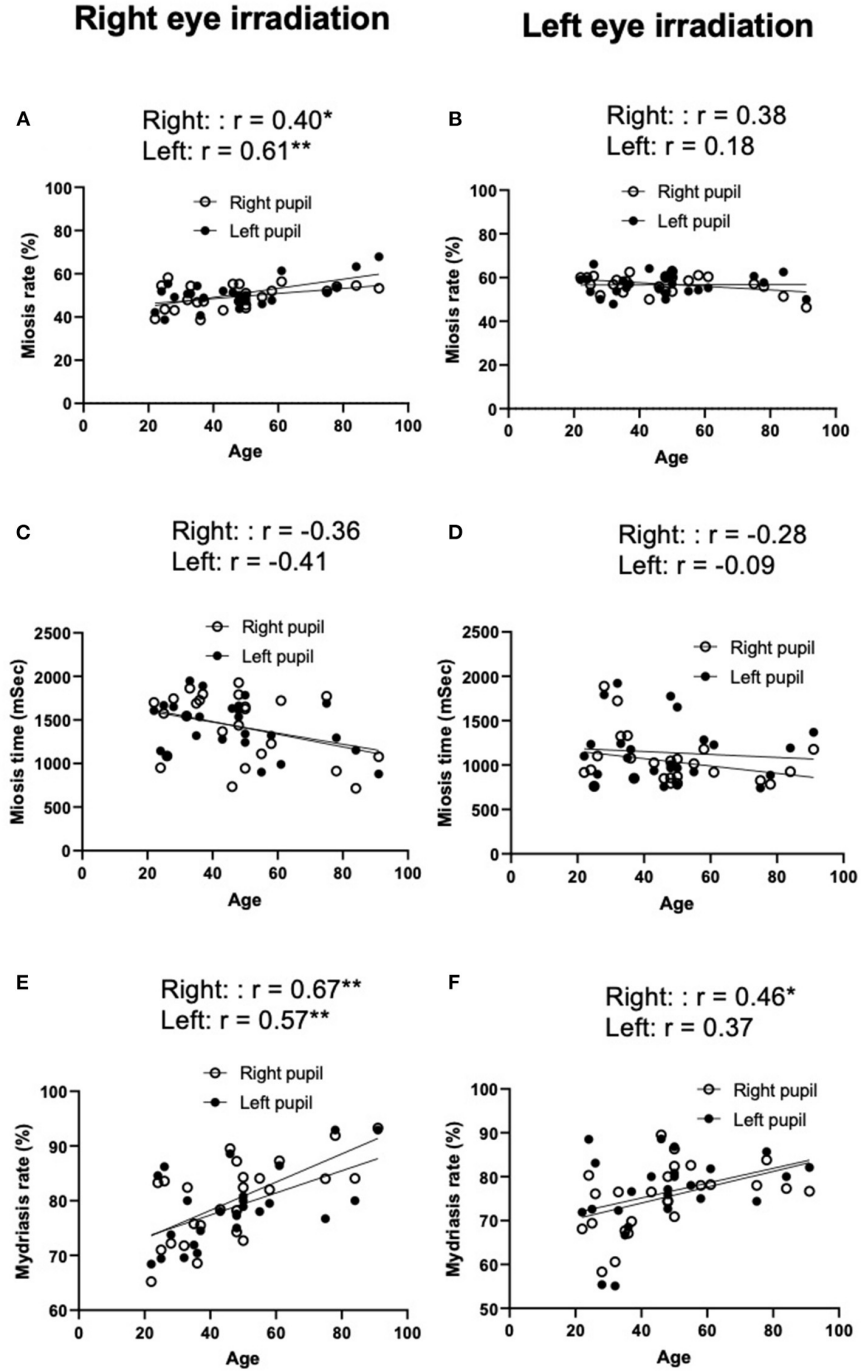
Relationship between age and miosis rate **(A,B)**, miosis time **(C,D)**, and mydriasis rate **(E,F)** in healthy volunteers. *r*, correlation coefficients. ^**^P < 0.01, ^*^P < 0.05. *N* = 25/group.

**Figure 6:**
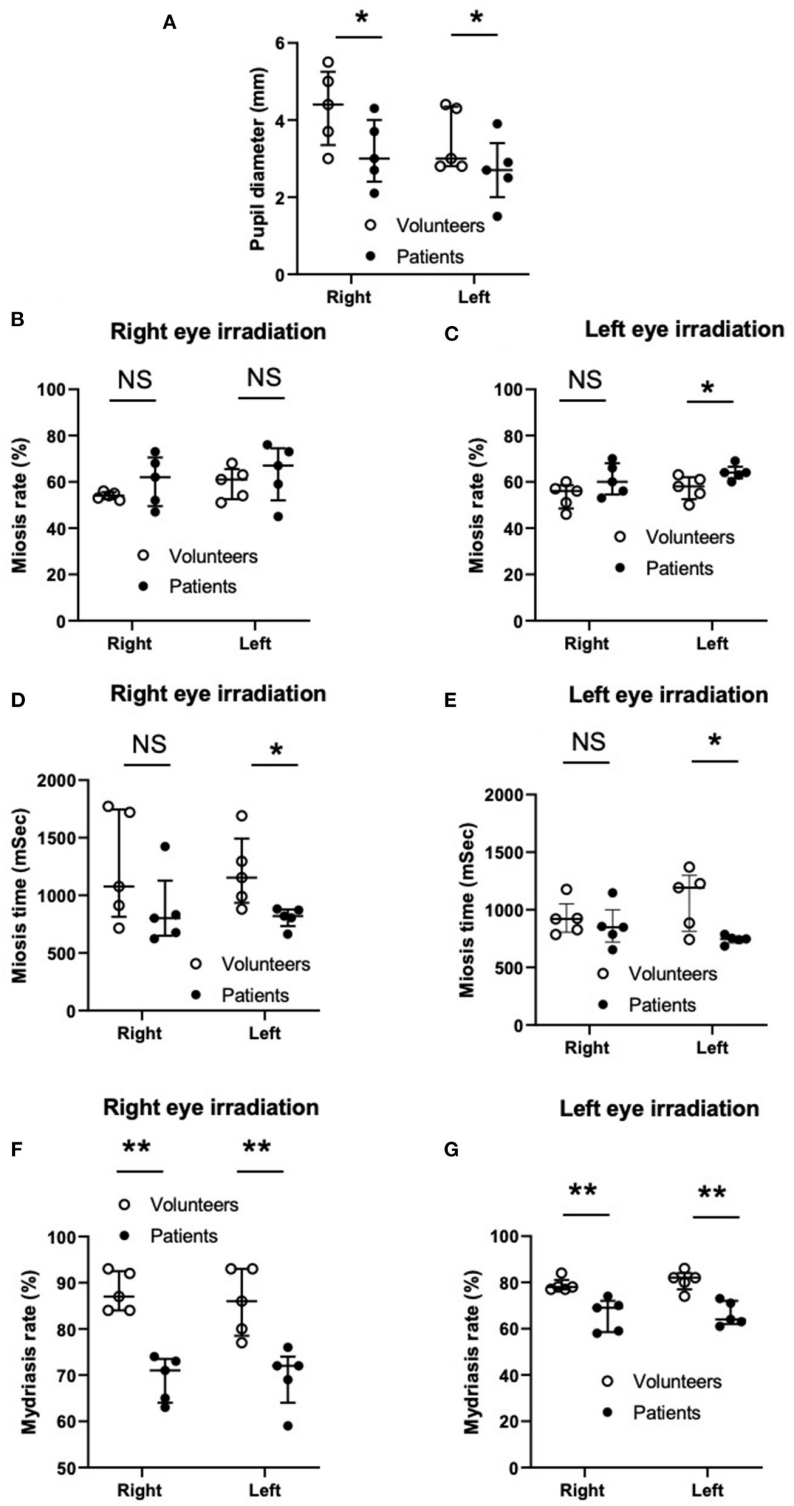
Comparison of spontaneous pupil diameters **(A)**, miosis rate **(B,C)**, miosis time **(D,E)**, and mydriasis rate **(F,G)** between the volunteers and patients. Column scatter plots representing the data distribution (open and closed circles), median (horizontal bar), and interquartile range (vertical bar). NS, not significant. ^**^P < 0.01, ^*^P < 0.05. *N* = 5 in each group.

Differences in continuous variables, including pupil diameter, miosis rate, miosis time, and mydriasis rate, between the two groups were compared using Student's *t*-test after first verifying the normal distribution of the data by the Shapiro–Wilk test; otherwise, the Mann–Whitney U-test was used. The associations between age and spontaneous pupil diameters, miosis rate, miosis time, and mydriasis rate in healthy volunteers were assessed with a correlation coefficient (*r*). All statistical analyses were performed using JMP^®^ 9 software (SAS Institute Inc., Cary, NC, USA). The column scatter plots shown in Figures 2–6 were generated using GraphPad Prism 8 (GraphPad Software, San Diego, CA, USA).

## Results

### Study 1

The spontaneous pupil diameters were significantly smaller in the left eyes than the right eyes (Figure 2). However, there were no significant differences in miosis rates (Figures 3A,B), miosis times (Figures 3C,D), and/or mydriasis rates (Figures 3E,F) in both the right and left pupils between direct and indirect irradiation.

### Study 2

There was a strong negative correlation between age and spontaneous pupil diameters (right: *r* = −0.70, *p* < 0.001 and left *r* = −0.81, *p* < 0.001: Figure 4). There was a moderate positive linear relationship between age and miosis rates in right eye irradiation (right, direct irradiation: *r* = 0.40, *p* < 0.05 and left, indirect irradiation: *r* = 0.61, *p* < 0.01, Figure 5A), but not in left eye irradiation (Figure 5B). As shown in Figures 5C,D, there were weak or negligible negative correlation between age and miosis time. There was a moderate positive correlation between age and mydriasis rates in right eye irradiation (right, direct irradiation: *r* = 0.67, *p* < 0.01 and left, indirect irradiation: *r* = 0.57, *p* < 0.01, Figure 5E). Similar tendencies were observed in left eye irradiation (right, indirect irradiation: *r* = 0.46, *p* < 0.05 and left, direct irradiation: *r* = 0.37, *P* = 0.07, Figure 5F). These results suggest that the mydriasis after irradiation was easier or earlier in the older groups. This may be due to the pupil diameters were smaller with increased age.

### Study 3

The average age was 74.0 ± 14.5 years old and 77.8 ± 10.0 years of age for the patient group and the volunteer group, respectively. The spontaneous pupil diameter without irradiation tended to be smaller in the left eye than in right eye in both the volunteer group and patient group (Figure 2), although this was not significant (Figure 6). There was no statistical difference in spontaneous pupil diameter between the volunteer group and the patient group in both the right and left eyes.

The miosis rate (Figures 6B,C), miosis time (Figures 6D,E), and mydriasis rate (Figures 6F,G) of the right and left pupils under right and left eye irradiation in the volunteer group and the patient group are shown in Figure 6A.

#### Miosis Rate

Under right eye irradiation (opposite side of the lesion in the patient group), the miosis rate of the right pupil (direct irradiation), and the left pupil (indirect irradiation) showed no significant difference between the volunteer group and the patient group. After left eye irradiation (side of lesion in the patient group), the miosis rate of the right pupil (indirect irradiation) also showed no difference between the volunteer group and the patient group. However, the miosis rate of the left pupil (direct irradiation) was significantly higher in the patient group than in the volunteer group, indicating that the pupil on the side of the lesion had more difficulty undergoing miosis.

#### Miosis Time

After right eye irradiation (opposite side of lesion in the patient group), the miosis time of the right pupil (direct irradiation) showed no difference between the volunteer group and the patient group. However, the miosis time of the left pupil (indirect irradiation) was significantly shorter in the patient group than in the volunteer group. After left eye irradiation (side of lesion in the patient group), the miosis time of the right pupil (indirect irradiation) showed no significant difference between the volunteer group and the patient group. However, the miosis time of the left pupil (direct irradiation) was significantly shorter in the patient group than in the volunteer group. These results indicated that the miosis response of the pupil on the side of the lesion in the patient group was quicker than that of the volunteer group, regardless of direct or indirect irradiation use.

#### Mydriasis Rate

After both right and left eye irradiation (both were on the opposite side of the lesion and lesion sites in the patient group), the mydriasis rates of both the right and left pupil (both direct and indirect irradiation) were significantly lower in the patient group than in the volunteer group, indicating that the mydriasis responses of the pupils on both the opposite and lesion site in the patient group were significantly more exaggerated than those in the volunteer group, irrespective of direct or indirect irradiation use.

## Discussion

The use of pupillometry has been limited by the lack of a uniform standard for assessment and the unavailability of easily obtained quantitative measurements of pupillary function (11). Although pupillometry has been accomplished with a penlight, variable factors, such as the amount of ambient light in the room, the observer's visual acuity, the distance of the penlight bulb from the patient's pupil, and the strength of the penlight batteries, could change the results of pupillometry measurements (1). Larson and Muhiudeen reported that routine clinical examinations performed with a penlight were unable to detect the presence of a pupillary light reflex when the light amplitude was <0.3 mm and the maximum constriction velocity was <1 mm/s (1). Several quantitative pupillometry devices have been developed since 1981, and a study using a quantitative pupilometer device has been reported (12). However, our study was novel, because the bilateral pupil diameter and pupillary light reflex were evaluated simultaneously and quantitatively using a mobile automatic pupillometer.

Regarding the anisocoria in healthy adults, Lam et al. reported that approximately 20% of normal adults had anisocoria of 0.4 mm or greater (13). They also reported that the number of adults with anisocoria increased with age, and anisocoria was seen in one-third of the normal adult group >60 years of age. In Study 1, the average age of the volunteers was younger than 60 years (47 years of age), and the healthy adults without intracranial lesions had anisocoria of approximately 0.7 mm. When we evaluated the anisocoria in the 27–39-year-old healthy volunteer group, many of them had anisocoria of approximately 0.5 mm. These results suggested that the anisocoria existed in healthy adults more than that previously reported (13). A literature search of the reason(s) why the right pupil diameter tended to be larger than the left one, however, found no studies.

Regarding miosis in healthy adults, it has been reported that the pupil diameter of the normal adult showed a miosis tendency that increased with age. This was called “Senile Miosis,” and it was hypothesized that the autonomic nervous system participated in the process (14). Specifically, it was suggested that the parasympathetic nerve function changes with age. However, there was no report examining the relationship of mydriasis rate, time, and miosis rate after light irradiation with age or the laterality of these parameters. The results in Study 2 showed significant relationships between miosis rates and age, as well as the laterality of this tendency. This further suggests that with the right eye irradiation it was more difficult to induce miosis, and it was easier to induce pupil dilation, in both the direct and indirect pupillary light reflexes, with age. However, most of the results of left eye irradiation did not show significant differences. If age-related parasympathetic nerve superiority has an influence on the pupillary light reflex, it should become easy to undergo miosis and harder to dilate with age. However, the results were not in agreement with this hypothesis. Generally, it was shown that the autonomic nerve function decreases with age for a long time (15, 16). Hence, it was suggested that the results from Study 2 (becoming easy to undergo miosis and hard to dilate with age) were not due to age-related parasympathetic nerve superiority, but to decreased autonomic nerve function, because the results of Study 1 suggested that the pupillary light reflex showed less physiological changes. In addition, our results suggested that the direct/indirect pupillary light reflex had greater accuracy than the pupil diameter, because the light reflex data did not reflect the change of physiological characteristics.

To our knowledge, this study was the first to present data regarding the indirect pupillary right reflex of patients with intracranial lesions in an ICU (Study 3). When comparing the patient group with the volunteer group, the pupil diameter did not show a significant difference between the volunteer group and the patient group in both the right and left eyes. However, the miosis times of the left pupils (i.e., the pupils on the side of the lesion) were significantly shorter in the patient group than in the volunteer group regardless of direct or indirect irradiation. One possible explanation may be following; in the patient group, the pupil diameters were smaller and the miosis rate of the left pupils were larger or tended to be larger (direct light reflex shown in Figure 6C, indirect light reflex shown in Figure 6B, respectively) indicating that substantial miosis distances were shorter. However, the mechanism(s) by which these phenomena were seen in the pupils only on the lesion sites needs to be further investigated. Furthermore, the mydriasis rate in both the left and right pupils (i.e., the pupils on both the opposite side and the same side of the lesion) were significantly lower in the patient group than in the volunteer group, indicating that the pupils of both sides of the patient groups were more exaggerated, irrespective of direct or indirect irradiation. These results suggested that we may be able to provide more meaningful information with not only pupil diameter but also the miosis time and mydriasis rate under direct and indirect irradiation. However, these direct and indirect light reflexes and the relationship between the light reflexes and the state of the disease need to be further investigated. The Hitomiru^®^ pupillometer is a novel, more convenient and useful device to measure pupil responses under indirect and indirect irradiation simultaneously, with digitally recorded results leading to faster measurement and accurate and objective assessments. In addition, because the instrument is a mobile type pupilometer, it can be easily transported to anywhere such as an ICU, emergency room, and pre-hospital care field.

Our study had several limitations. The time interval from the first measurement (light irradiation for the right side) to the next measurement (light irradiation for the left side) was only 2 s. The pupil diameters in many subjects may not return to baseline during the 2-s time interval just before the beginning of left eye irradiation. Therefore, the results of the left eye irradiation should be different from the results obtained when the time interval is longer than 2 s or when the left eye irradiation is done first. Second, although we selected patients with intracranial lesions on only the left side, the type of disease, onset, and severity varied. Third, volunteers did not check precise ophthalmologic examinations before starting this experiment even though they had neither intracranial lesions nor eye lesions based on the hearing investigation. Fourth, mydriasis rates of left pupils at the end of 2-s visible light off after right eye irradiation, which was the beginning of left eye irradiation, were <100% in both volunteers and patients, indicating that 2-s interval was not enough for the pupils to return to the baseline. This might affect the measurements during left eye irradiation. Finally, because we only examined Japanese patients, these findings may not translate to patients of other ethnicities or races.

In conclusion, our studies showed that healthy adults may have approximately 0.5 mm anisocoria, miosis was harder, and mydriasis was easier with increased age, and there were differences in miosis rates, miosis times, and mydriasis rates between the healthy adult and the patient groups with intracranial lesions under both direct irradiation and indirect irradiation. The Hitomiru^®^ pupillometer, a newly developed instrument used to measure pupil responses under direct and indirect irradiation, simultaneously and digitally recorded the data, and could be a useful device to further investigate the relationship between pupil reflexes and the state of intracranial diseases.

## Data Availability Statement

The original contributions presented in the study are included in the article/supplementary material, further inquiries can be directed to the corresponding author/s.

## Ethics Statement

The studies involving human participants were reviewed and approved by The Institutional Review Board of Hyogo College of Medicine. The patients/participants provided their written informed consent to participate in this study. Written informed consent was obtained from the individual(s) for the publication of any potentially identifiable images or data included in this article.

## Author Contributions

JK: direction of the study and editing the manuscript. HN: practice of the study. IY, AM, and NM: acquisition and analysis of data for the work. YO: substantial contributions to the conception or design of the work and analysis and interpretation of data for the work. All authors contributed to the article and approved the submitted version.

## Conflict of Interest

The authors declare that the research was conducted in the absence of any commercial or financial relationships that could be construed as a potential conflict of interest.

## Publisher's Note

All claims expressed in this article are solely those of the authors and do not necessarily represent those of their affiliated organizations, or those of the publisher, the editors and the reviewers. Any product that may be evaluated in this article, or claim that may be made by its manufacturer, is not guaranteed or endorsed by the publisher.
